# Combined Cytotoxic Effects of Carvacrol-Based Essential Oil Formulations

**DOI:** 10.3390/plants15020211

**Published:** 2026-01-09

**Authors:** Öykü Gönül Geyik, İmren Hasoğlu, Ayşe Simay Metin, Selin Aktar Kiremitci

**Affiliations:** 1 Medical Biology Department, Faculty of Medicine, İstinye University, 34408 Istanbul, Türkiye; oyku.geyik@istinye.edu.tr; 2 Medical Biology and Genetics Doctoral Program, Institute of Graduate Education, İstinye University, 34408 Istanbul, Türkiye; 3 Pharmaceutical Botany Department, Faculty of Pharmacy, İstinye University, 34408 Istanbul, Türkiye

**Keywords:** carvacrol, cell viability, breast cancer, triple negative, thyme

## Abstract

Carvacrol, a phenolic monoterpene predominantly found in *Origanum* species, has been reported to exhibit antimicrobial, anti-inflammatory, antioxidant, and cytotoxic effects. Formulations such as Vacrol and S-Mix, enriched with carvacrol and complementary essential oil compounds, may enhance therapeutic efficacy while reducing toxicity. Essential oil components were analyzed via GC-MS. Cell viability was assessed using the sulforhodamine B (SRB) assay at different concentrations and incubation periods. An in ovo chorioallantoic membrane (CAM) assay was performed to investigate tumor volume changes and histopathological alterations. Vacrol and S-Mix demonstrated concentration- and time-dependent cell viability-attenuating effects in MDA-MB-231 cells, with significant reductions in viability at higher concentrations (1–10 mM). In ovo, S-Mix induced ~40% reduction in tumor volume and promoted apoptotic morphology compared to controls. Combined effects of carvacrol with α-pinene, eugenol, and β-terpineol likely contributed to enhanced bioactivity. These findings support further preclinical and mechanistic investigations to validate their therapeutic potential.

## 1. Introduction

Triple-negative breast cancer (TNBC) is an aggressive subtype of breast cancer characterized by the absence of estrogen receptor, progesterone receptor, and HER2 expression. Owing to its high metastatic potential, chemoresistance, and lack of targeted treatment options, TNBC remains one of the most challenging breast cancer subtypes to manage clinically [1,2,3]. These limitations have intensified the search for new therapeutic candidates, particularly multi-targeted natural compounds capable of modulating several oncogenic pathways simultaneously.

Essential oils and their phenolic monoterpenes have gained increasing attention in oncology due to their broad pharmacological properties, including antimicrobial, antioxidant, anti-inflammatory, and cytotoxic activities. Carvacrol (5-isopropyl-2-methylphenol), a predominant component of *Origanum onites*, *Origanum vulgare*, *Thymus vulgaris*, *Thymus zygis*, and *Satureja hortensis*, is one of the most extensively studied essential oil constituents for its anticancer potential [4,5,6,7]. Its antimicrobial and antioxidant activities are mechanistically linked to its cytotoxic effects: carvacrol disrupts lipid membrane integrity, alters ion gradients, induces oxidative imbalance, and triggers mitochondrial dysfunction—mechanisms that similarly promote apoptosis in cancer cells through increased ROS generation, cytochrome-c release, and caspase activation [8,9,10].

Carvacrol has demonstrated antiproliferative effects across multiple breast cancer models, with IC_50_ values reported between 50 and 200 μM in MCF-7 and MDA-MB-231 cells [10,11,12,13]. Despite this, monotherapy with carvacrol presents important limitations. Achieving strong cytotoxic responses often requires relatively high concentrations, which may increase the risk of off-target toxicity. Furthermore, its physicochemical properties—such as rapid membrane diffusion, volatility, and limited intracellular retention—may reduce efficacy in complex tumor microenvironments.

To overcome these limitations, strategies have been proposed that combine carvacrol with other monoterpenes or sesquiterpenes. Compounds such as thymol, α-pinene, eugenol, β-terpineol, and camphene have been shown to enhance membrane permeability, modulate redox signaling, potentiate ROS-mediated cell death, and amplify caspase activation, collectively lowering IC_50_ thresholds to 20–80 μM in TNBC models [14,15]. These findings support the concept that essential oil synergy, rather than isolated single-molecule activity, may provide a more potent and physiologically relevant anticancer approach.

Based on this rationale, standardized carvacrol-based formulations such as Vacrol and S-Mix were developed. Both originate from thyme oil-rich species and contain carvacrol as the primary component, yet differ in their complementary monoterpene profiles. Vacrol contains a higher proportion of carvacrol, whereas S-Mix includes a broader spectrum of terpenes such as α-pinene, eugenol, β-bisabolene, cinnamaldehyde, and β-terpineol. These formulations aim to enhance therapeutic efficacy by leveraging multicomponent interaction, improving membrane penetration, amplifying apoptotic signaling, and potentially reducing the concentration of carvacrol required to achieve cytotoxic effects.

In the context of TNBC—where treatment options are limited, targeted therapies are lacking, and chemoresistance is frequent—such multi-targeted natural formulations offer a promising research direction. However, direct comparative evidence on the cytotoxic potency of carvacrol-based mixtures remains scarce, particularly in preclinical breast cancer models.

Therefore, this study investigates the cytotoxic effects of Vacrol and S-Mix using in vitro (MDA-MB-231 and MCF-12A) and in ovo CAM models, examining concentration- and time-dependent cell viability inhibition, and changes in tumor morphology and volume. By evaluating these formulations within a mechanistic framework of carvacrol combination, the study aims to determine whether multicomponent essential oil formulations exhibit superior cytotoxic activity compared with carvacrol-dominant preparations, thereby providing foundational evidence for their potential use as complementary agents against triple-negative breast cancer.

## 2. Results

### 2.1. GC-MS Analysis Results

**Figure 1:**
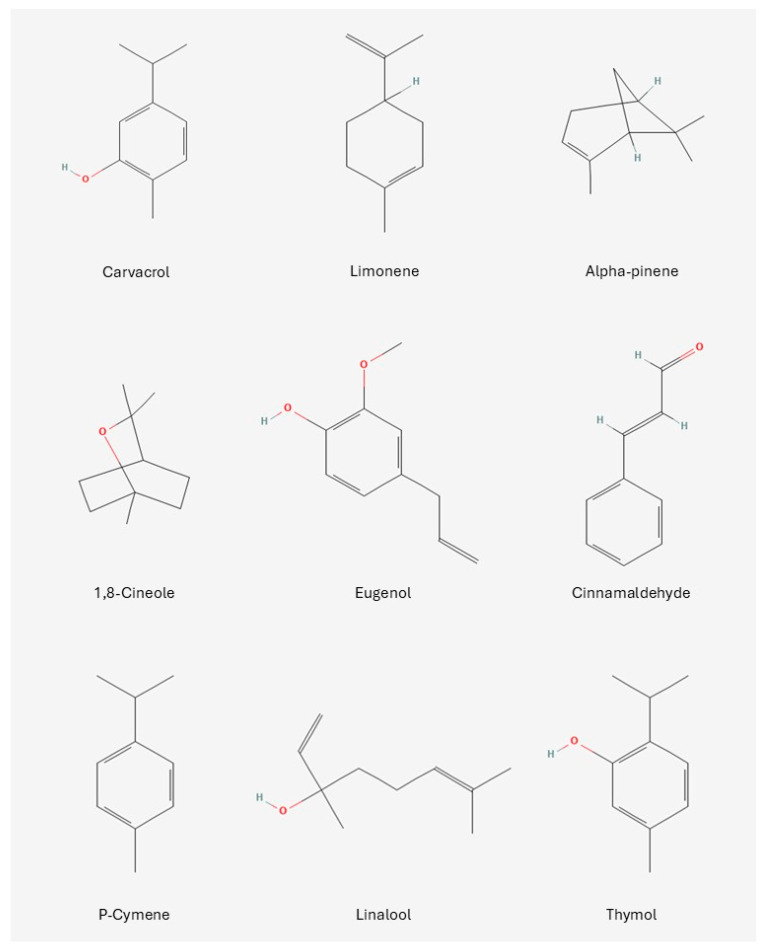
Structural formulas of the most prominent compounds found in Vacrol and S-Mix.

The essential oil components of Vacrol and S-Mix were analyzed by GC-MS and are shown in Table 1. Among these components, the most prominent ones are given in Figure 1.

### 2.2. In Vitro Analysis Results

**Figure 2:**
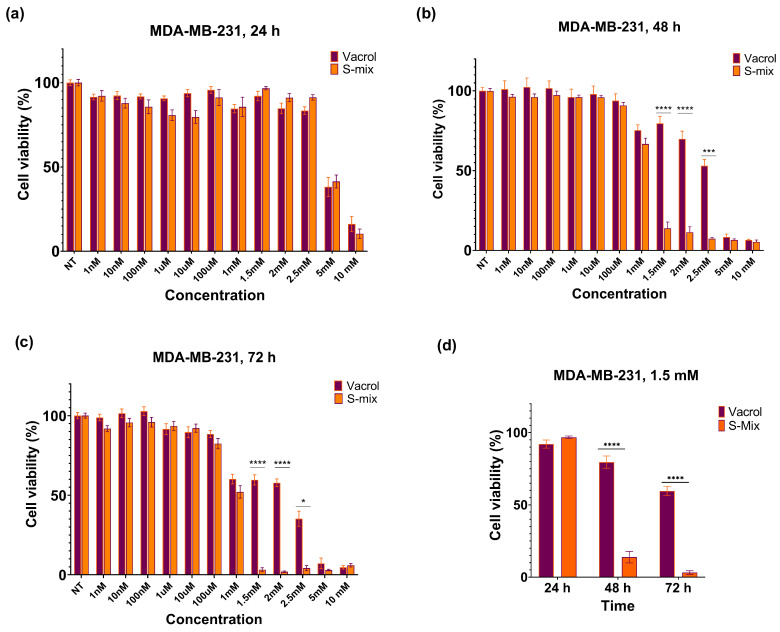
Effects of S-Mix and Vacrol on the viability of MDA-MB-231 breast cancer cells after treatment for (**a**) 24 h, (**b**) 48 h, and (**c**) 72 h. (**d**) Effects of S-Mix and Vacrol on the viability of MDA-MB-231 breast cancer cells after 24, 48, and 72 h of treatment with 1.5 mM concentration. Data are presented as mean ± SD of 3 biological and 6 technical replicates (n = 3). Significant differences are indicated by asterisks (* *p* = 0.0222, *** *p* = 0.0001, **** *p* < 0.0001; Kruskal–Wallis followed by Dunn’s multiple-comparison test).

Cell viability assays after Vacrol and S-Mix treatments in the MDA-MB-231 cell line were performed at 24, 48, and 72 h (3 biological replicates, 18 technical replicates) and are shown in Figure 2.

**Figure 3:**
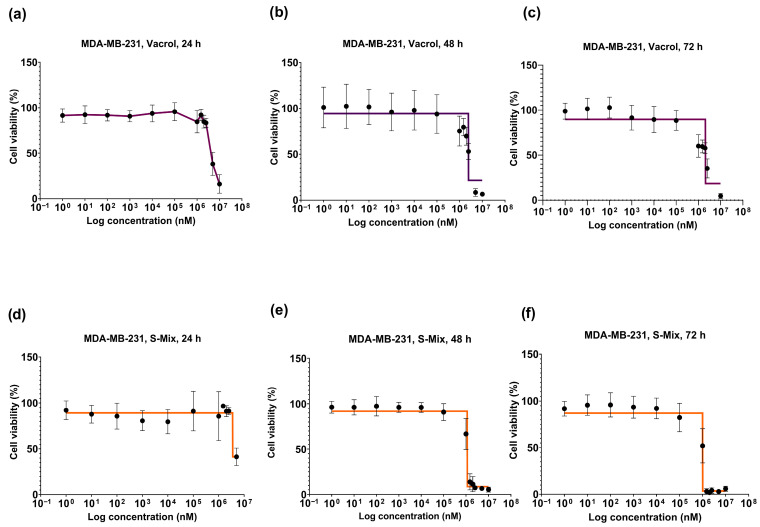
Concentration- and time-dependent viability graphs of MDA-MB-231 cells after treatment with S-Mix and Vacrol for 24, 48, and 72 h. Vacrol (**a**–**c**), S-Mix (**d**–**f**). Data are presented as mean ± SD of 3 biological and 6 technical replicates (n = 3); nonlinear regression analysis. The data are shown as log_10_ values.

At 48 h, a clear reduction in cell viability was observed at higher concentrations (≥1.5 mM) with S-Mix compared to Vacrol (*** *p* = 0.0001, **** *p* < 0.0001). These results indicate time-dependent cytotoxic and/or cytostatic effects. At lower concentrations, cell viability remained above 90% and showed a concentration-dependent pattern. At 72 h, the effects on cell viability became more evident. S-Mix was highly effective even at low concentrations (* *p* = 0.0222, **** *p* < 0.0001). At 1.5 mM, S-Mix reduced cell viability to 3.15%, while Vacrol showed 60.11% viability at the same concentration (Figure 2d). At the 48 h timepoint, the IC_50_ values were calculated as 3.2 mM for Vacrol and 1.1 mM for S-Mix, and at 72 h, the IC_50_ values were 2.4 mM for Vacrol and 1 mM for S-Mix (Figure 3).

**Figure 4:**
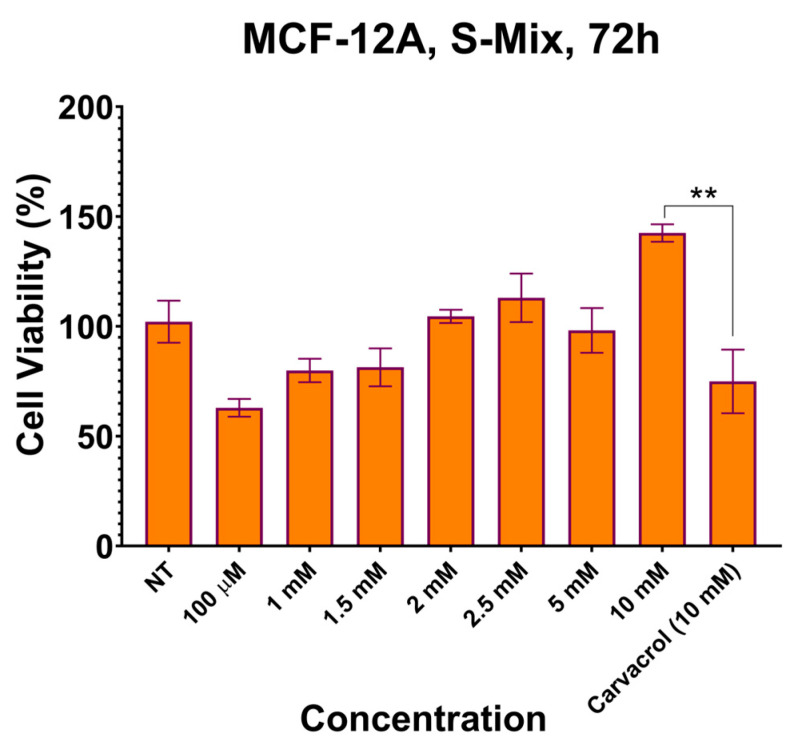
Effects of S-mix on the viability of MCF-12A human breast epithelial cells after treatment for 72 h. Data are presented as mean ± SD of 3 biological and 6 technical replicates (n = 3). Significant differences are indicated by asterisks (** *p* = 0.0097; Kruskal–Wallis followed by Dunn’s multiple-comparison test).

Given that S-Mix was more effective than Vacrol in the MDA-MB-231 cell line, its effect on the MCF-12A mammary epithelial cell line was examined (**** *p* < 0.0001). The concentrations that were effective in the MDA-MB-231 TNBC cell line were applied to the MCF-12A mammary epithelial cell line for 72 h. Carvacrol, the main component of S-Mix in the MCF-12A cell line, was administered as a positive control in the amount contained in the highest concentration of S-Mix (10 mM). While the IC_50_ value of S-Mix was observed at a concentration of 1 mM in the MDA-MB-231 TNBC cell line, the cell viability was observed to be 80% at the same concentration in the MCF-12A mammary epithelial cell line (Figure 4).

**Figure 5:**
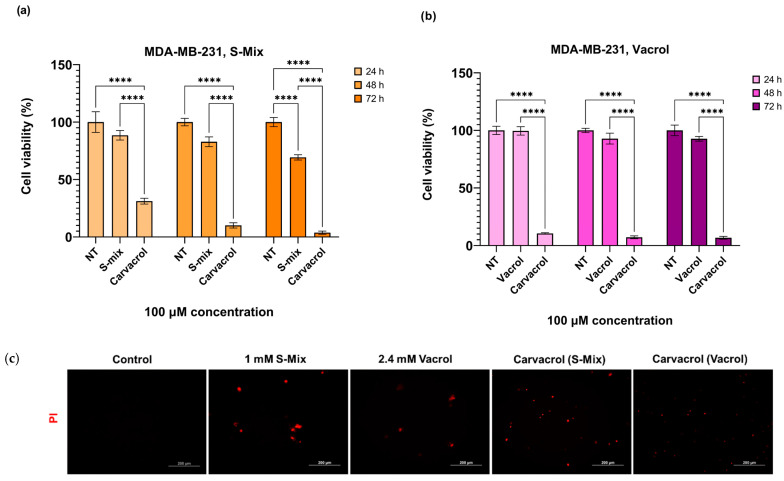
Comparative effects of S-Mix, Vacrol, and carvacrol on MDA-MB-231 cell viability and PI positivity: (**a**,**b**) The effect of 100 μM S-Mix, Vacrol, and Carvacrol treatment on cell viability in the MDA-MB-231 cell line for 24, 48, and 72 h. Data are presented as mean ± SD of 3 biological and 6 technical replicates (n = 3). Significant differences are indicated by asterisks (**** *p* < 0.0001; Kruskal–Wallis followed by Dunn’s multiple-comparison test). (**c**) Fluorescence microscopy analysis of cell viability loss following S-Mix, Vacrol, and Carvacrol treatment for 72 h in MDA-MB-231 cells. After treatment for 72 h, cells were stained with 1 µg/mL PI for 30 min, and the cells were then imaged using fluorescence microscopy (Ex./Em.:493/635 nm). Red fluorescence indicates PI-positive (dead) cells. Scale bar 200 μm.

Carvacrol, the main component of S-Mix and Vacrol, was applied to MDA-MB-231 cells at a concentration of 100 µM for 24, 48, and 72 h. Since cell viability dropped below 30% following the 100 µM Carvacrol treatment used as a positive control, higher concentrations were not tested. These results demonstrate that carvacrol reduces cell viability more effectively in cancer cells compared to S-Mix and Vacrol (Figure 5a,b). PI staining results revealed that the S-Mix, Vacrol, and Carvacrol treatments induced a marked increase in red fluorescent signals relative to the control, indicating enhanced cell death. Among the treatments, the S-Mix group exhibited the highest level of PI positivity. Consistent with the PI staining results, representative brightfield images revealed distinct morphological signs of cell death, such as cell shrinkage, cytoplasmic fragmentation, and loss of membrane integrity in the treatment groups. These morphological alterations were most prominent in the S-Mix group, correlating with the high intensity of PI-positive signals (Figure 5c).

These findings show that S-Mix has stronger viability-inhibiting effects on MDA-MB-231 cells compared to Vacrol. The lower IC_50_ values of S-Mix, together with its ability to reduce viability at lower concentrations and shorter incubation times, suggest that it effectively inhibits cancer cell growth. On the other hand, Vacrol was active at higher concentrations and longer treatment times, but it was not as strong as S-Mix. Overall, these results indicate that S-Mix may be a more promising cytotoxic agent than Vacrol, with viability-inhibiting activity that increases in a concentration- and time-dependent manner.

### 2.3. In Ovo Analysis Results

In this study, the response of MDA-MB-231 (triple-negative breast cancer) cells to Vacrol (V) and S-Mix (S) treatment was evaluated in the in ovo CAM model at the end of a 72-h treatment period, using both macroscopic and histopathological analyses. The aim of the study was to comparatively assess the effects of both formulations on tumor growth, cell morphology, and apoptosis. Ranjan et al. (2023) demonstrated that the CAM assay is a reliable model for investigating breast cancer biology, particularly angiogenesis and invasion [16].

**Figure 6:**
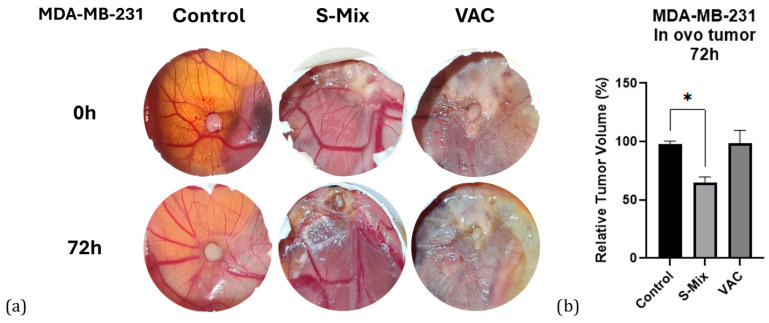
(**a**) Macroscopic appearance of tumors. In ovo images of CAM tumors from the control, Vacrol, and S-Mix groups at treatment initiation (t_0_) and at 72 h (t_72_). (**b**) Tumor volume changes. Percentage values of tumor volumes at 72 h (t_72_), normalized to baseline (t_0_), based on measurements performed with ImageJ. Volumes were calculated using the formula width × length^2^. Data are presented as mean ± SD of 3 biological replicates per group (n = 3). * *p* < 0.05; Kruskal–Wallis followed by Dunn’s multiple-comparison test.

Macroscopic tumor volume analysis was performed with ImageJ ver.1.54p software using photographs taken at the start of treatment (t_0_) and at 72 h (t_72_). First, the scale in each egg photograph was normalized using a fixed reference point (e.g., a shell fracture line). Then, the length and width of each tumor were measured, scale differences were normalized, and proportional volumes were calculated using the formula: width × length^2^. For each tumor, the baseline t_0_ volume was set to 100, and the t_72_ values were expressed as percentage changes. Inflammatory-cell infiltration appeared more prominent in Vacrol-treated tumors, whereas apoptosis-like morphology (e.g., nuclear condensation/fragmentation) was more frequently observed in S-Mix-treated tumors; however, apoptosis was not quantified in this study. As a result of the analysis, tumor volume in the control group remained approximately stable (~100%), no significant change was observed in the Vacrol group, while in the S-Mix group, tumor volume decreased by about 40% (t_0_ = 100 → t_72_ ≈ 60) (Figure 6).

**Figure 7:**
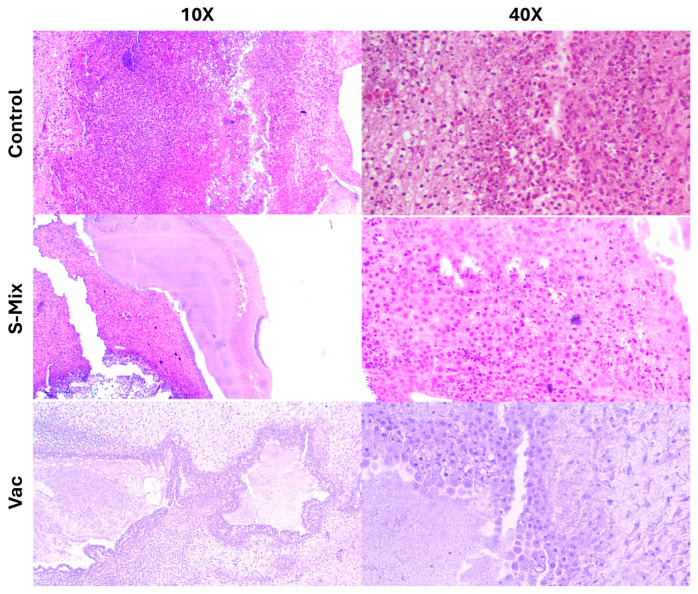
Histopathological examination images of in ovo CAM tissue following Vacrol and S-Mix administration.

Histopathological evaluations revealed a large number of tumor cells in the control group that heavily infiltrated the Matrigel matrix and spread into the CAM (chorioallantoic membrane) tissue. The morphological integrity was preserved, and the infiltration density was significantly higher compared to the treatment groups. In the Vacrol group, the Matrigel structure was not observed in the tumor area. Instead, cells likely belonging to the CAM and a significant infiltration of inflammatory cells were seen. However, indicators of apoptosis were found to be at a limited level. In the S-Mix group, a high density of tumor cells was found within the Matrigel. The cell nuclei were clearly visible, and nuclear fragmentation was observed in some areas. Compared to the Vacrol group, a higher number of cells suggesting apoptosis were detected in the S-Mix application (Figure 7).

These findings indicate that both formulations induced structural changes in the tumor microenvironment, but the macroscopic volume reduction and apoptosis were more pronounced in the S-Mix group. The data obtained from the in ovo model suggest that the anti-tumor potential of these formulations may materialize through different mechanisms.

## 3. Discussion

In this study, Vacrol and S-Mix were shown to induce time- and concentration-dependent viability-suppressing effects on the MDA-MB-231 cell line. The reduction in cell viability was particularly pronounced after 72 h of incubation and at higher concentrations, supporting the cytotoxic potential of these formulations.

The component analysis results of the formulations are in line with this observation from a biological perspective. The high carvacrol content (50.1% in Vacrol; 24.00% in S-Mix) is considered the primary contributor to the pro-apoptotic, antiproliferative, and anti-inflammatory activities described in the literature [9,11,14].

Our in vitro viability analyses clearly demonstrated the effects of S-Mix and Vacrol on TNBC cells. After 24 h of incubation, both compounds caused a significant reduction in cell viability at high concentrations (5 mM and 10 mM). However, at 48 h, S-Mix induced a pronounced decrease in cell viability even at lower concentrations compared to Vacrol. At this timepoint, the IC_50_ value for S-Mix was determined to be 1.1 mM, whereas cells treated with Vacrol at the same concentration retained over 50% viability. Similarly, following 72 h of incubation, S-Mix continued to reduce cell viability at lower concentrations, while Vacrol required higher concentrations to achieve a comparable effect. Additionally, S-Mix, which was more effective than Vacrol in the MDA-MB-231 cell line, showed 80% cell viability at a concentration of 1 mM (IC_50_) in MCF-12A mammary epithelial cells. Carvacrol, even when used as a positive control, reduced the viability of MDA-MB-231 cancer cells below 30% at a concentration of 100 µM; thus, higher concentrations were not tested. Our SRB assays determined that S-Mix had a more potent effect on cell viability compared to Vacrol in a time- and dose-dependent manner. This suggests that, in addition to the strong effect of the compound Carvacrol alone, the S-Mix formulation provides a more successful means than Vacrol that may reflect formulation-dependent interactions. These findings raise the possibility that S-Mix has a selective effect on cancer cells. Indeed, S-Mix contains a higher concentration of compounds such as α-pinene, camphene, and eugenol that are reported to increase cell membrane permeability, facilitating the entry of carvacrol into target cells and producing synergistic antimicrobial and anticancer effects [17,18,19]. To complement the SRB viability readout, we performed propidium iodide staining after 72 h exposure, which showed increased PI-positive cells in the treatment groups, consistent with loss of membrane integrity and increased terminal cell death. Importantly, PI staining does not distinguish apoptotic from necrotic modes of cell death; therefore, mechanistic conclusions regarding apoptosis require dedicated assays (e.g., Annexin V/cleaved caspases).

These findings suggest that S-Mix exerts its effects at an earlier timepoint, whereas Vacrol produces a similar response only after prolonged exposure. Although carvacrol is the primary active component of both formulations, the rapid response observed with S-Mix cannot be solely attributed to this molecule. S-Mix also contains additional constituents, including cinnamon oil (cinnamaldehyde) and peppermint oil (menthol/menthone), which may contribute to combined effects with carvacrol to modulate cellular processes. Indeed, cinnamaldehyde has been reported to induce apoptosis and autophagy in both hematologic and solid tumor cell lines [20,21,22] and to regulate tumor growth and progression [23,24]. Similarly, menthol has been shown to suppress proliferation and exhibit anticancer properties in various cancer cell lines [25] and to exert antiproliferative effects in leukemia cells [26]. Furthermore, menthol and menthone, the major constituents of peppermint (*Mentha piperita*) oil, display cytotoxic, anti-inflammatory, and antioxidant activities that enhance their efficacy against cancer cells [27]. On the other hand, we cannot say this is definitely due to synergistic interaction since we did not experiment each component one by one with each other to be able to draw isolobolograms or perform the Chou–Talalay method. Therefore, we can only claim that these mixtures show combined effects exhibited by the components in them. This is the main limitation of our work, and we plan to do these experiments to illuminate the mechanism in the future.

Similar effects have been documented with other natural essential oil combinations. For example, an in vivo study of *Origanum onites* oil demonstrated an 84–85% reduction in tumor volume, an effect attributed to its high carvacrol content [28]. Likewise, carvacrol has been shown in preclinical studies to exert antiproliferative activity in MCF-7 and MDA-MB-231 breast cancer cell lines by arresting the cell cycle in the G_0_/G_1_ phase and enhancing caspase-3 activation [11,29].

Arunasree (2009) reported that 100 µM carvacrol reached IC_50_ after 48 h in MDA-MB-231 cells and induced apoptotic morphology [30]. In line with this, our results showed that ~150–160 µM (1 mM stock-derived equivalent) treatment for 72 h reduced viability below 60%. Li et al. (2020) demonstrated that carvacrol regulates cell cycle progression via the TRPM7 channel and significantly induces apoptosis at 200 µM [12]. This mechanism may underlie our observed decreases in viability, with TRPM7 inhibition leading to G0/G1 arrest and transition to apoptosis [12]. In MCF-7 cells, carvacrol has been reported to inhibit the PI3K/AKT pathway, halting the cell cycle and inducing apoptosis. Since MDA-MB-231 cells are triple-negative, disruption of similar oncogenic pathways may explain their sensitivity to carvacrol. Furthermore, in vivo glioblastoma studies showed that carvacrol reduces tumor growth by suppressing Akt/GSK3β signaling, indicating systemic anticancer activity.

In our in ovo analyses, the proportional tumor volume reduction (t_0_ = 100 → t_72_ ≈ 60) and the morphology favoring nuclear fragmentation/apoptosis observed with the S-Mix application in the MDA-MB-231 CAM model point to the multi-level modulation of tumor biology by its monoterpene content, particularly those components that act in the same direction as α-terpineol. α-Terpineol is a natural monoterpene that can reduce tumor cell proliferation and facilitate apoptosis by suppressing NF-κB signaling. Its concentration-dependent inhibition of NF-κB’s nuclear translocation and activity has been experimentally demonstrated before [31]. In MDA-MB-231 cells, carvacrol has been shown to induce apoptosis via the mitochondrial pathway, confirmed by a decrease in membrane potential, cytochrome-c release, caspase activation, and PARP cleavage in a concentration-dependent manner [30]. Our results are also consistent with the recent study by Meijer et al. (2024), which validated the CAM assay as a reliable and reproducible model for testing natural compounds, including essential oils, in oncology research [32].

The difference favoring S-Mix that our findings highlight can be explained by the combination effect created by the co-occurrence of monoterpenes with similar effects (e.g., terpinen-4-ol, α-pinene, etc.), rather than by the effect of α-terpineol alone. Indeed, terpinen-4-ol has been shown to increase apoptosis in various tumor cell lines (including MDA-MB-231) and can enhance the effects when combined with targeted agents. This suggests that the combined use of monoterpene pools can more easily overcome the apoptotic threshold [33].

Collectively, these data suggest that the early and pronounced effect of S-Mix is likely due not only to carvacrol but also to the combined interaction among its additional bioactive constituents, which together modulate cellular responses. In this context, S-Mix elicits a faster and more effective response, whereas Vacrol achieves a similar outcome only cumulatively, requiring prolonged exposure.

One of the novel aspects of this work is that Vacrol and S-Mix are not composed solely of carvacrol but also contain other essential oil constituents. According to analysis reports SM-D-001 and VL-D-001, S-Mix contains 24.00% carvacrol, along with 1,8-cineole (11.7%), α-pinene (12.7%), eugenol (7.6%), and thymol (0.5%). The Vacrol formulation, on the other hand, in addition to containing 50.1% carvacrol, also includes eucalyptol (9.7%), α-terpinolene (1.3%), isomenthol (2.26%), and eugenol (4.7%). Each of these components has been described in the literature for its anticancer, antioxidant, and anti-inflammatory properties. In particular, eugenol is known for its ability to induce apoptotic cell death. Thymol and α-pinene can increase cell membrane permeability, thereby enhancing cytotoxic effects. β-terpineol has been reported to exert cell cycle–arresting effects.

Although detailed pathway analyses were beyond the scope of the present study, the biological effects observed here are consistent with previously reported activities of carvacrol when combined with other bioactive terpenoids in cancer cell models. Multiple independent studies have demonstrated that carvacrol-containing combinations can modulate apoptosis, oxidative stress, cell cycle control, and mitochondrial integrity in vitro [34,35]. For instance, carvacrol in combination with thymol has been shown to increase caspase-3 activation and intracellular ROS while downregulating Bcl-2 in MCF-7 breast cancer cells, indicating apoptosis induction through mitochondrial pathways [14,36]. Similar synergistic anticancer effects have been reported for other monoterpene and sesquiterpene combinations—such as limonene with β-caryophyllene in A549 lung cancer cells [37] and linalool with geraniol in MDA-MB-231 cells—where alterations in mitochondrial membrane potential, apoptotic indices, and cell-cycle arrest were documented [17]. Importantly, these studies relied on mechanistic assays that were not employed in the present work. Accordingly, while the current findings support an antiproliferative effect at the cellular and histopathological levels, any reference to signaling pathways such as NF-κB, PI3K/AKT, or ion-channel-associated mechanisms should be interpreted as literature-supported context rather than direct evidence [38,39]. Future studies will therefore focus on validating the molecular mechanisms of the newly described formulations through targeted analyses, including protein, transcript, and oxidative stress measurements [18].

Therefore, the fact that Vacrol and S-Mix contain not only carvacrol but also these additional constituents suggests that the observed reduction in cell viability may be stronger than the effect of carvacrol alone. Such combined action may enable these formulations to exhibit significant biological activity even at lower concentrations. The presence of essential oil components suggests that Vacrol and S-Mix may be used pharmacologically at lower, potentially less toxic doses while maintaining efficacy. This finding supports their potential application, particularly in multi-targeted treatment strategies and combination therapies. Verification of these effects through advanced mechanistic and in vivo studies will be essential for translation into clinical applications.

## 4. Materials and Methods

### 4.1. GC-MS Analysis

The analysis of thyme (*Origanum onites*) essential oils and volatile compounds (Vacrol^®^ and S-Mix^®^ were purchased from Carmed Pharmaceuticals, Istanbul, Türkiye) was performed using a Thermo TSQ—GC-MS system and evaluated according to TLM-05-G3OK-05-55. A 5-MS capillary column (30 m length, 0.25 mm internal diameter, 0.25 µm film thickness) was used. The analysis was performed by the Scientific and Technological Research Council of Türkiye (TÜBİTAK, Kocaeli, Türkiye), the National Metrology Institute (UME, Kocaeli, Türkiye); a certified reference material (UME CRM 1301, Kocaeli, Türkiye) was used as the reference material. This CRM was employed to determine the percentages of related compounds in vacrol by carrying out the peak area normalization method.

Helium was employed as the carrier gas at a constant flow rate of 0.8 mL/min. The oven temperature program was as follows: initial hold at 50 °C for 5 min, followed by an increase of 3 °C/min to 240 °C. The split ratio was set to 40:1, while HS-SPME applications were performed with splitless injections. The injector temperature was adjusted to 250 °C. The mass spectrometer operated in electron impact (EI) mode at 70 eV ionization energy, scanning a range of 35–450 *m*/*z*.

Volatile oil components were identified by comparing the obtained mass spectra with Wiley and NIST libraries. Retention times (RTs) of the components were measured. Major compounds were confirmed by co-injection with appropriate standard substances. Retention indices (RI) were calculated relative to a homologous series of n-alkanes (C8–C30) analyzed under identical GC–MS conditions according to the Van den Dool and Kratz equation. RI (Lit.) values were obtained from the NIST Chemistry WebBook and Adams, 2007 [40]. Relative contents (% *w*/*w*) were calculated using the area normalization method.

### 4.2. In Vitro Analyses

#### 4.2.1. Cell Culture

The MDA-MB-231 (CRM-HTB-26, ATCC, Manassas, VA, USA) cell line was used as the experimental model. MDA-MB-231 is an aggressive human triple-negative breast cancer (TNBC) model commonly used to evaluate the efficacy of novel therapeutic agents due to its lack of hormonal receptors and resistance to conventional treatments. In this study, the MDA-MB-231 cells were cultured in DMEM High Glucose supplemented with 10% FBS, 1% L-glutamine, and 1% penicillin/streptomycin and the mammary epithelial cells MCF-12A (CRL-3598, ATCC, Manassas, VA, USA) were cultured in DMEM-F12 medium supplemented with 0.5 mg/mL hydrocortisone, 10 µg/mL insulin, 5% horse serum, 20 ng/mL human EGF, and 1% penicillin/streptomycin incubated in humidified 5% CO_2_ incubator at 37 °C [41,42].

#### 4.2.2. Cell Viability Assay

The effects of Vacrol and S-Mix, applied at increasing logarithmic concentrations, were evaluated for their impact on cell viability. Experiments were conducted with incubation periods of 24, 48, and 72 h. The effect of Vacrol (V) and S-Mix (S) on cell viability was determined using the sulforhodamine B (SRB) assay. MDA-MB-231 and MCF-12A cells were seeded into 96-well plates in 100 µL of medium at densities 5 × 10^3^ cells/well, 3 × 10^3^ cells/well, and 2 × 10^3^ cells/well for 24-, 48-, and 72-h timepoints, respectively.

After 24 h of incubation, cells were treated with V and S at concentrations of 1 nM, 10 nM, 100 nM, 1 µM, 10 µM, 100 µM, 1 mM, 2 mM, 2.5 mM, 5 mM, and 10 mM. Carvacrol was used at 100 µM in MDA-MB-231; at 10 mM in MCF-12A (matching the highest S-Mix carvacrol-equivalent) as a positive control. Stock solutions were prepared in complete cell culture medium based on Trolox equivalents (V: 10.848 mM, S: 4.236 mM). Cells were incubated for 24, 48, or 72 h, and then fixed with 50% (*w*/*v*) trichloroacetic acid (TCA). Following fixation, TCA was removed with distilled water, and SRB solution was added and incubated in the dark. Excess SRB was removed with 1% acetic acid, and the plates were air-dried. Bound dye was solubilized with 150 µL/well of 10 mM Tris base (pH 10.0). Absorbance was measured at 564 nm using a spectrophotometer (LUMIStar Omega, BMG Labtech, Ortenberg, Germany). Each condition was performed with 6 technical replicates and 3 biological replicates. The results obtained were analyzed both graphically and statistically by GraphPad Prism v.9 software, using the non-parametric ANOVA method and Kruskal–Wallis test to perform multi-group comparisons, followed by Dunn’s post hoc test. The significance level was accepted as *p* < 0.05 at the 95% CI. Experimental data are presented as mean ± SD.

#### 4.2.3. Propidium Iodide Staining

MDA-MB-231 cells were seeded in 6-well plates (5 × 10^4^ cells/well). Following exposure of cells to 1 mM S-Mix, 2.4 mM Vacrol and equivalent Carvacrol contained in each formulation for 72 h, cell death was determined by 1 µg/mL Propidium Iodide (PI) (#HY-D0815 MedChem Express, Monmouth Junction, NJ, USA, Ex./Em.:493/635 nm) for 30 min and the cells were subsequently visualized using fluorescence microscopy at 20× magnification (Olympus, Tokyo, Japan).

### 4.3. In Ovo Analyses

#### 4.3.1. Incubation Process

Fertilized Ross 308 chicken eggs were incubated at 37 °C with 60% humidity until egg development day (EDD) 1. At EDD1, small holes were drilled into the lateral and lower (air sac-containing) parts of the shell to allow air entry and membrane detachment. The eggs were placed in the incubator with the pointed end facing upward.

At EDD4, a 1–2 cm circular window was opened on the upper surface of the eggshell, sealed with adhesive tape, and returned to the incubator. Embryo viability was monitored daily.

#### 4.3.2. Histopathological Analysis

Tumor volume measurement and histopathologic analysis were then performed. On EDD7, MDA-MB-231 breast cancer cells (1 × 10^6^) were mixed with Matrigel and applied onto the chorioallantoic membrane (CAM). On EDD10, 50 µL of Vacrol or S-Mix, calculated based on Trolox values and diluted accordingly in HBSS, was applied directly onto the tumors, which were photographed. On EDD13, tumors were re-photographed, excised from the CAM, and fixed in 10% buffered formalin [16,43]. Fixed samples were processed in a semi-open carousel-type tissue processor. Tissues were dehydrated in graded alcohols (50%, 70%, 80%, 96%, and 100%), cleared with xylene, and embedded in paraffin at 58–60 °C. Paraffin blocks were sectioned at 3–5 µm using a rotary microtome (CUT 5062, Slee, Nieder-Olm, Germany). Sections were stained with hematoxylin and eosin (H&E) for histopathological evaluation. The tumor volume data were analyzed both graphically and statistically by GraphPad Prism v.9 software, using the non-parametric ANOVA method and Kruskal–Wallis test to perform multi-group comparisons, followed by Dunn’s post hoc test. The significance level was accepted as *p* < 0.05 at the 95% CI. Experimental data are presented as mean ± SD.

## 5. Conclusions

Vacrol and S-Mix are plant-derived, carvacrol-containing essential-oil formulations that were chemically profiled by GC–MS and evaluated in vitro (SRB assay) and in ovo (CAM xenograft) in a triple-negative breast cancer model. Both formulations reduced MDA-MB-231 cell viability in a time- and concentration-dependent manner, with S-Mix showing greater potency under the tested conditions. In the in ovo CAM model, S-Mix was associated with an approximately 40% reduction in tumor volume over 72 h and histopathological features consistent with increased cell injury and apoptosis-like morphology compared to controls, whereas Vacrol showed limited macroscopic volume reduction with distinct tissue-level changes. Consistent with the viability data, PI staining provided an additional, morphology-linked indicator of treatment-associated terminal cell death, but does not by itself establish an apoptotic mechanism.

While formal synergy quantification (e.g., CI/isobologram analyses) and molecular pathway validation were beyond the scope of the present study, the observed activity profiles across complementary models are consistent with component-driven effects and provide a solid rationale for targeted mechanistic and synergy-focused follow-up experiments.

Future studies should include component-level combination testing, expanded breast cancer panels, and pharmacologically grounded dosing/PK–toxicity evaluation to assess translational feasibility.

## Figures and Tables

**Table 1:** The contents of Vacrol and S-Mix were determined by the GC-MS method.

Component	RI (Exp.)	RI (Lit.)	Vacrol (% *w*/*w*)	RT_Vacrol_ (min)	S-Mix (% *w*/*w*)	RT_S-Mix_ (min)
α-Pinene	939	939	3.5	4.30	12.7	4.33
Camphene	954	954	0.4	5.30	0.5	5.29
β-Pinene	979	979	0.7	6.41	1.3	6.41
Sabinene	975	975	–	–	0.7	6.83
δ-3-Carene	1011	1011	0.4	7.65	1.5	7.66
Myrcene	991	991	0.3	8.21	0.5	8.21
α-Terpinene	1017	1017	0.3	8.59	–	–
Limonene	1031	1031	0.8	9.17	13.0	9.24
1,8-Cineole	1033	1033	9.7	9.42	11.7	9.44
δ-Terpinene	1062	1062	1.1	10.64	0.7	10.66
p-Cymene	1024	1024	3.2	11.38	3.4	11.37
α-Thujone	1114	1114	1.3	15.59	1.5	15.59
β-Thujone	1124	1124	0.4	16.11	0.4	16.11
Menthone	1155	1155	0.8	16.68	0.9	16.70
Camphor	1146	1146	1.2	18.03	1.4	18.03
Linalool	1095	1095	6.3	19.06	2.3	19.04
Caryophyllene	1418	1418	0.9	20.07	0.9	20.07
4-Terpineol	1177	1177	1.3	20.31	0.8	20.31
Menthol	1167	1167	1.9	21.22	2.2	21.22
Humulene	1454	1454	0.3	21.76	0.4	21.76
Terpinenyl acetate	1349	1349	0.8	22.44	1.1	22.44
Fenchyl alcohol	1118	1118	0.6	22.55	0.4	22.55
Borneol	1165	1165	0.9	22.65	0.4	22.65
Bisabolene	1505	1505	1.1	23.14	0.7	23.13
Cinnamaldehyde	1272	1272	4.3	29.71	7.1	29.72
Eugenol	1356	1356	4.7	32.17	7.6	32.17
Eugenol acetate	1521	1521	–	–	0.3	33.72
Thymol	1290	1290	2.1	32.60	0.5	32.60
Carvacrol	1300	1298–1300	50.1	33.16	24.0	33.13
9-Octadecenoic acid	2086	2086	–	–	0.5	49.49
Hexadecadienoic acid, methyl ester	2095	2095	0.6	51.10	0.6	51.10

Values ± 0.1, RT: retention time, RI: retention index, Exp.: experimental, Lit.: literature.

## Data Availability

The datasets used and/or analysed during the current study are available from the corresponding author upon reasonable request.

## Abbreviations

The following abbreviations are used in this manuscript:

SRB	Sulforhodamine B
CAM	Chorioallantoic Membrane
TNBC	Triple-Negative Breast Cancer
EDD	Egg Development Day
